# Role of landscape context in *Toxoplasma gondii* infection of invasive definitive and intermediate hosts on a World Heritage Island

**DOI:** 10.1016/j.ijppaw.2022.08.010

**Published:** 2022-08-29

**Authors:** Sono Okada, Yuki Shoshi, Yasuhiro Takashima, Chizu Sanjoba, Yuya Watari, Tadashi Miyashita

**Affiliations:** a Laboratory of Biodiversity Science, Graduate School of Agricultural and Life Sciences, The University of Tokyo, 1-1-1, Yayoi, Bunkyo-ku, Tokyo, 113- 8657, Japan; b Laboratory of Molecular Immunology, Graduate School of Agricultural and Life Sciences, The University of Tokyo, 1-1-1 Yayoi, Bunkyo-ku, Tokyo, 113-8657, Japan; c Department of Veterinary Parasitological Diseases, Faculty of Applied Biological Science, Gifu University, 1-1 Yanagido, Gifu, 501-1193, Japan; d Forestry and Forest Products Research Institute, Matsunosato 1, Tsukuba, Ibaraki, 305-8687, Japan

**Keywords:** Feeding cats, Infectious disease, Landscape analysis, One health, Transmission, Zoonoses

## Abstract

Free-ranging cats are invasive species threatening biodiversity worldwide. They may also impose an environmental risk to humans and livestock through the transmission of zoonotic diseases. We investigated antibody levels against *Toxoplasma gondii* in free-ranging cats and black rats (definitive and representative intermediate hosts) by ELISA and determined their relationships with landscape environmental factors on Tokunoshima Island, Japan, the Natural World Heritage site. We found a higher seroprevalence (>70%) in both cats and black rats in landscapes where the cattle barn density was high. This was consistent with higher density of rats revealed in our trapping survey. The spatial scale of landscape factors affecting infection was broader in cats (1 km buffer radius) than in black rats (100 m buffer radius). Both cats and rats showed an increasing trend in optical density (OD) values with increasing body weight and landscape cattle barn density, suggesting that the antibody concentration increases as the chance of exposure to *T. gondii* in the environment increases. Thus, management actions to stop humans from feeding cats and to control rat populations without using cats are both necessary to reduce the human health risk as well as to conserve endangered species on the island.

## Highlights

• High seroprevalence of *T. gondii* was found in cats and black rats on an island.
• Seroprevalence was high (>70%) in landscapes where there were many cattle barns.
• Antibody level in cats was lower in forests and higher around residential areas.
• Spatial scale of environmental factors affecting infection was broader in cats.

## Graphical abstract

**Figure:**
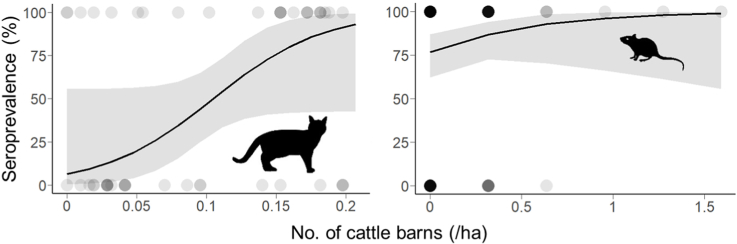

## 1 Introduction

Invasive species are major drivers of biodiversity loss and threaten invaded ecosystems through predation, competition, and the physical alteration of ecosystems (Jeschke et al., 2014; Russell and Blackburn, 2017). In addition, owing to increases in zoonotic diseases (Jones et al., 2008), understanding the role of invasive species in introducing and transmitting new pathogens is important for the health of entire ecosystems, including humans.

The domestic cat (*Felis silvestris catus*) is a typical invasive species. Owing to their highly predatory and adaptive nature, cats living outdoors have had a tremendous negative impact on ecosystems (Medina et al., 2011; Nogales et al., 2013; Doherty et al., 2016), driving 33 species to extinction worldwide (Medina et al., 2011), thereby, listed in the 100 of the World's Worst Invasive Alien Species (Lowe et al., 2000). By contact with both humans and wildlife, cats act as a reservoir for zoonotic diseases, such as rabies, toxoplasmosis, cat scratch disease, and plague, thereby posing a public health threat (Dubey, 2008; Gerhold and Jessup, 2012).

Among cat-caused diseases, toxoplasmosis caused by *Toxoplasma gondii* is a common disease whose only definitive host is domestic cats in regions without native felines (Khan and Khan, 2018). *T. gondii* infects almost all warm-blooded animals as intermediate hosts (Dubey, 2008) and poses a risk of infection to livestock and humans, in addition to wildlife. When humans are infected, serious symptoms may occur in fetuses, newborns, and immunocompromised patients (Khan and Khan, 2018; Fard et al., 2020). Recently, infections have been linked to mental illness, suicide, and traffic accidents (Flegr et al., 2002; Torrey and Yolken, 2003). Since no vaccine in human is currently available (Wang et al., 2019), it is important to identify regions with a high risk of infection to prevent the spread of toxoplasmosis (Kijlstra and Jongert, 2008; Dabritz and Conrad, 2010).

There are three main routes of *T. gondii* transmission to intermediate hosts: the ingestion of oocysts that flowed into the soil and drinking water from definitive host feces, ingestion of cysts in infected prey animals, and vertical transmission from mothers (Dunn et al., 1999). The prevalence of oocyst shedding is particularly high in cats experimentally infected with bradyzoites of cysts (Dubey and Frenkel, 1976; Dubey, 2009). Therefore, the sexual reproduction cycle, which is completed by the predation of small animals (e.g., passerine birds and rodents infected by feeding on soil-attached foods containing oocysts) is an important process in the spread of *T. gondii*. As most animals in the wild inhabit spatially heterogeneous environments, environmental risk assessment for *T. gondii* infection should focus on the spatial distribution of infection status of cats (definitive hosts) and small animals (intermediate hosts). There have been numerous studies that examined antibody prevalence in various animals (see Dubey (2010) for review). However, few studies have simultaneously investigated the spatial distribution of *T. gondii* antibody prevalence in cats and small animals and their correspondence with environmental factors from a landscape perspective (but see Ballash et al. (2015) for exception). For analyzing landscape factors, not only landscape composition but also spatial scale (extent) should be identified (Benton et al., 2003; Miyashita et al., 2012). Although no studies have ever explored the spatial scale affecting *T. gondii* infection, such knowledge should provide valuable implication for the spatial risk assessment.

In this study, we evaluated antibody levels against *T. gondii* in free-ranging cats and black rats (*Rattus rattus*) on Tokunoshima Island (southeastern Japan) to determine how infection with *T. gondii* is associated with environmental factors. The prevalence of serum antibodies to *T. gondii* in cats on Tokunoshima Island is reported to be as high as 47.2% (Shoshi et al., 2021). The landscape of this island is composed mainly of forests, agricultural lands, and residential areas, a typical heterogeneous landscape in Asian countries. About 30% of the residents of Tokunoshima are engaged in primary industry (Agriculture, 2019), and cattle barns are widespread on this island. In other countries, *T. gondii* antibody prevalence in cats and wild animals tended to be elevated in and around livestock farms (Lehmann et al., 2003; Simon et al., 2017, 2018), but the simultaneous estimation for each species group in the same region is generally lacking. Also, as both free-ranging cats and black rats generally prefer forests and residential areas to agricultural lands (cats: Doherty et al., 2014; black rats: Hathaway and Blackmore, 1981; Feng and Himsworth, 2013), these land-cover types may be related to the antibody prevalence in both species.

The main hypotheses addressed in this study are: (1) antibody prevalence is high in landscapes with high density of cattle barns, and with large areas of forests or residence while less areas of agriculture lands, (2) spatial scale influencing infection is larger in cats than in rats due to a larger home range in cats. As this island harbors many endangered species (such as Amami rabbits and Tokunoshima spiny rat; e.g. Maeda et al., 2019) that are preyed upon by free-ranging cats, such knowledge would provide important implications for cat management aiming to reduce risk for biodiversity and human health.

## 2 Materials and methods

### 2.1 Study site

**Fig. 1:**
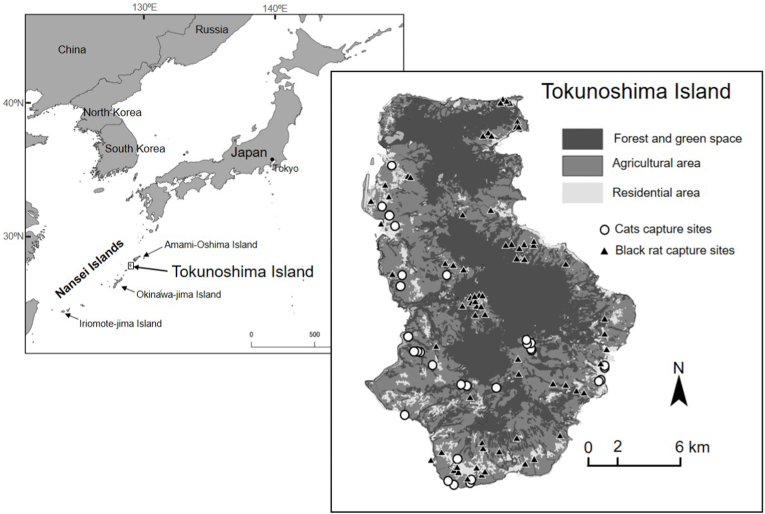
Map showing the study area. Open circles and black triangles in the map indicate, respectively, capture sites of cats and black rats.

Tokunoshima Island is located in the Nansei Islands, southwestern Japan (27°45′N, 128°58′E), with an area of 248 km^2^ (Fig. 1). The island has a warm subtropical climate throughout the year, with an average annual temperature of about 21.6 °C and annual precipitation of about 1912 mm (Kagoshima Prefecture, 2019). About 43% of the island is covered with forest, and there are mountains with peaks of 500–600 m in the central and northern parts of the island (Kagoshima Prefecture, 2019). In the forest, evergreen oaks, such as *Quercus miyagii* and *Castanopsis sieboldii*, are dominant, and endemic and endangered species, such as Amami rabbits (*Pentalagus furnessi*), Tokunoshima spiny rat (*Tokudaia tokunoshimaensis*), and Ryukyu long-furred rat (*Diplothrix legata*), are found. Owing to this unique ecosystem, Tokunoshima Island was registered as a Natural World Heritage site in 2021, together with the adjacent Amami-Oshima Island, Okinawa-Jima Island, and Iriomote-Jima Island (UNESCO World Heritage Center, 2021).

The human population on Tokunoshima Island is about 24,000 (Kagoshima Prefecture, 2019). The core industry is agriculture, with a cultivated area of 6890 ha, consisting mainly of sugar cane fields (Kagoshima Prefecture, 2019). Beef cattle production is also common; there are about 1000 beef cattle barns, with 9.6 breeding cattle per household, on average (Agriculture, 2019). Cats are often kept outside around barns with the main purpose of controlling black rats (Kazato et al., 2020; Qi et al., 2022), which are believed to attract venomous snakes (*Protobothrops flavoviridis*), a threat to residents. A questionnaire survey revealed that cats are fed by humans in 45% of barns on the island (Kazato et al., 2020).

### 2.2 Sample collection

Blood samples of free-ranging cats were provided from individuals captured in 2017 in the cat management program conducted by local governments (Amagi Town, Tokunoshima Town, and Isen Town), in which captured cats were taken to a veterinary clinic for infertility and castration, and blood sampling. For each individual cat, the capture point, capture date, and body weight were recorded. Black rats were captured by the authors and blood samples were collected in 2019 and 2020. Cage traps (W14.5 × D22.5 × H10 cm) with peanuts as baits were set in farmlands (sugarcane field or pasture field), green spaces (small woodlots in agricultural and residential landscapes), and residential areas throughout Tokunoshima Island. Each trap was patrolled daily. Captured rats were weighed and blood samples were collected. For each trap, the total number of days installed and the number of rats caught were recorded. Serum was separated from blood samples and stored at −20 °C until further analysis. Notice that 128 out of 155 were used for blood sampling (see Results).

### 2.3 Enzyme-linked immunosorbent assay

Cat serum anti-*T. gondii* antibody was measured by an indirect ELISA previously (Shoshi et al., 2021), following the method of Can et al. (2014). Briefly, 96-well microtiter plates were coated with an antigen suspension prepared from 3.0 × 10^5^/ml samples of *T. gondii* RH Ankara strain tachyzoites (Delibas et al., 2006). After the plates were blocked with 1% bovine serum albumin (BSA), cat serum samples were diluted 1:100 and added to the wells. Next, plates were incubated with 1:20,000-diluted Goat anti-cat IgG horseradish peroxidase (HRP)-conjugated antibodies (Life Technologies, Frederick, MD), then reacted with a substrate (TMB Microwell Peroxidase Substrate System; SeraCare Life Sciences, Milford, MA). After the reaction was stopped by the addition of 50 μl of 2[N] H_2_SO_4_ per well, absorbance was measured at 450 nm by a microplate reader (SpectraMax Paradigm; Molecular Devices, Sunnyvale, CA).

Rat serum anti-*T. gondii* antibody was also measured by ELISA. Crude tachyzoite lysate was prepared from *T. gondii* PLK strain as described previously (Appiah-Kwarteng et al., 2019). Then, each well of a 96-well microtiter plate was coated with 100 μl of 10 μg/ml antigen suspension. After the plates were blocked with 1% BSA in PBS-T, 1:100-diluted rat serum samples were applied at 100 μl per well for 1 h. Next, plates were incubated with 100 μl of 1:20,000-diluted goat anti-rat IgG HRP-conjugated antibodies (Life Technologies) for 1 h and reacted with the substrate (SeraCare Life Sciences). Reactions were stopped by the addition of 50 μl of 2[N] H_2_SO_4_ per well. Absorbance was measured at 450 nm by a microplate reader (Molecular Devices).

Ten serum samples from specific pathogen-free (SPF) cats and 56 serum samples from conventionally raised rats of Wistar-Imamichi strain were used as negative controls (NC) (Supplementary S3). All samples were analyzed in duplicates, and the optical density (OD) vales were calculated by the mean of each sample. The cutoff value was set to the mean OD value for the NC plus three standard deviations for both cats and rats analysis of ELISA.

### 2.4 Environmental variables

To extract landscape variables around the capture points of cats and black rats, the area proportions of forest, agricultural land, residential area, and barn density were calculated within the buffer generated around each capture point. Land use data were obtained from the National Land Numerical Information (Ministry of Land, Infrastructure, Transport and Tourism, Japan) based on RapidEye satellite images taken in 2016. The minimum size of the land use data is 100 m mesh, with 12 classes of land cover types. We used three land cover classes (agricultural lands, forests, residential areas) for the analysis, because they occupy most of the area on Tokunoshima island and are likely to affect the distribution of cats and black rats (see Introduction). Barn data were provided by the local government of Tokunoshima Island.

Different buffer sizes were set for cats and black rats because they have different home range size. For cats, buffer radii of 500 m and 1 km were generated, because the home range of feral cats ranges mostly from 100 to 500ha (Konecny, 1987; Biró et al., 2004; McGregor et al., 2015). For black rats, radii of 100 m, 200 m, and 500 m were generated, because the home range is generally less than 10 ha (Pryde et al., 2005; Byers et al., 2019). Although the 500 m buffer size is clearly larger than the ordinal home range of black rats, it was included to allow comparison with the spatial scale of cats.

Landscape indices for each buffer were calculated using QGIS 3.10.0. Since the areas of forest, agricultural land, and residential area were correlated, principal component analysis (PCA) was performed to reduce these into two variables (1st and 2nd PCA axes, hereafter PC1 and PC2).

Spatial autocorrelation was calculated to account for the spatial independence of the capture points. Moran's eigenvector maps (MEM), which describe synthetic spatial patterns of study sites in a two-dimensional space, are often used for multiple regression or canonical analysis to account for spatial dependence (or spatial filtering) and to infer large scale environmental factors affecting spatial gradients (Dray et al., 2006). MEM axes are automatically extracted from large-to fine-scale patterns, and each spatial pattern is quantified by the eigenvalue of the MEM axis (Legendre and Legendre, 2012). MEMs were constructed using the coordinate data for study sites, and Delaunay triangular and MEM scores were calculated (Legendre and Legendre, 2012), and the vector values of the MEM were calculated. The R package “adespatial” was used for MEM calculation.

### 2.5 Statistical analysis

To evaluate environmental factors affecting *T. gondii* infection, two indices were used as response variables: seropositivity based on the cutoff value and the OD value itself. The OD value itself was also used as an index of *T. gondii* infection to avoid information loss due to the cutoff. OD values were used as a measure of infection frequency because the concentration of antibodies against *T. gondii* in wild boars decreases with time elapsed since infection (Opsteegh et al., 2011) and a high antibody concentration in cats could be a result of environmental exposure or repeated contact with the parasites (Afonso et al., 2006).

A generalized linear model (GLM) was constructed to investigate the relationship between antibody prevalence or OD values and landscape variables. The explanatory variables were PC1 and PC2 of landscape variables, barn density, body weight, and MEM axes. Here, five MEM axes were used for cats and 10 axes were used for black rats, reflecting the larger number of samples for black rats. In the GLM of OD, the logarithmic link and gamma distribution were used for cats, and the identity link and normal distribution were used for black rats, as determined by the visual inspection of data dispersion. In the seroprevalence analysis, the logit link and binomial distribution were used.

Since samples from cats and rats were obtained by different methods, with different sampling locations and spatial resolutions, it was not possible to directly compare the antibody prevalence or concentrations between the two species. However, as the spatial ranges of capture sites for the two species overlapped largely (Fig. 1), separate analyses for the spatial distributions of infected cats and rats are likely to reveal the similarity and difference of the environmental factors influencing infection risks of *T. gondii* for the two species.

The spatial scale of landscape factors, which may affect antibody levels in animals, was estimated by searching the best model. We first created a group of models consisting of all possible combinations of explanatory variables for each buffer size and calculated the Akaike information criterion (AIC). The model with the lowest AIC for each buffer size was extracted, and then the model with the lowest AIC globally across different buffer sizes was identified as the best spatial scale. The Nagelkerke's *R*^2^ was also calculated for the best models at each spatial scale. Furthermore, to account for uncertainty arising from choosing the best model, the model-averaged parameter estimate was calculated for each variable, using models with ΔAIC <2. This is because a set of models having ΔAIC <2 are considered competing models in terms of performance (Burnham and Anderson, 2002). Variables for which the 95% CI of parameter estimates did not overlap with zero were considered influential.

To investigate the difference in the relative density of black rats among habitat types (agricultural land, green space, and residential area), the capture rate (number of captures/number of trap-days) was calculated. Fisher's exact test was used to detect significant differences in capture rate between habitat types.

All statistical analyses were performed using R for Windows 3.6.1 (R Core Team, 2019).

## 3 Results

### 3.1 Seroprevalence

Blood samples were collected from 49 cats and 128 black rats (Fig. 1). The cutoff values for cats and black rats were, respectively, 0.438 and 0.0564. Samples with OD values above the cutoff values were considered as positive samples. Each of the seroprevalence was 49.0% for cats and 69.5% for black rats.

### 3.2 Spatial scale and landscape PCA

The landscape spatial scale that best explained the seroprevalence or OD values of cats was determined by the model AICs obtained at a 500 m or 1 km buffer size. The model for seroprevalence had a lower AIC value at a 1 km scale, implying that the landscape elements at this scale were influential (Table 1). The ΔAIC (difference in AICs) between 500 m and 1 km models was about 3, and ΔAIC between 1 km and the null model was greater than 15 (Table 1), showing that the best model was superior. For OD values, the model with a 1 km buffer scale clearly had a lower AIC than that of the 500 m scale (ΔAIC >7), which had much lower AIC than that of the null model (ΔAIC >35) (Table 1). This indicates that the performance of model for OD values was greater than that of the seroprevalence model. The Nagelkerke's *R*^2^ was 0.555 for the best OD value model and 0.664 for the best seroprevalence model, indicating fairly high explanatory power.

**Table 1:** The AIC value of the best generalized linear model (GLM) explaining the spatial variation of seroprevalence or optical density (OD) values at each buffer size for cats and black rats. Null models are the models with only intercept. Bold values indicate the global best model across scales.

	Cats			Black rats			
	Buffer size		Null model	Buffer size			Null model
	500 m	1 km		100 m	200 m	500 m	
Seroprevalence	57.6	**54.7**	69.9	**114.4**	116.5	116.7	159.4
OD value	43.6	**35.9**	66.4	**−370**	−359.4	−358.4	−321.1

For black rats, model performance was compared between three spatial scales. The AIC value for the seroprevalence model was lowest at 100 m (Table 1). The ΔAIC values between the 100 m model and the 200 and 500 m models were greater than 2, and the ΔAIC between the 100 m model and the null model was nearly 40 (Table 1). The best model for the OD value of black rats was also at the buffer scale of 100 m. The ΔAIC values between models at 100 m and other scales were greater than 10, and the ΔAIC value between the 100 m model and the null model was nearly 50 (Table 1). The Nagelkerke's *R*^2^ was 0.405 for the best OD value model and 0.549 for the best seroprevalence model, indicating fairly high explanatory power.

In summary, the landscape spatial scale influencing seroprevalence and OD values differed markedly between cats and black rats (i.e., radii of 1 km and 100 m).

The PC1 axis of land use for the 1 km buffer, identified as the best scale for cats, had a positive loading for forests and a negative loading for residential areas. The PC2 axis for this scale had a positive loading for agricultural area (Table 2). On the other hand, the PC1 axis for land use at the 100 m buffer, the best scale for black rats, showed a negative loading for agricultural area. The PC2 axis showed a positive loading for residential area and a negative loading for forests (Table 2). Note that details and interpretation of MEM axes were not described here, since they were included as covariates rather than focal variables.

**Table 2:** Factor loadings of each landscape element that constitutes principal component axes, PC1 and PC2, for the analyses of cats and black rats. Note that buffer size is 1 km for cats and 100 m for back rats.

Landscape elements	Cats		Black rats	
	PC1	PC2	PC1	PC2
Agricultural area	0.17	0.872	−0.791	0.087
Forests	0.654	−0.448	0.342	−0.758
Residential area	−0.737	−0.198	0.507	0.647

### 3.3 Factors affecting infection

**Fig. 2:**
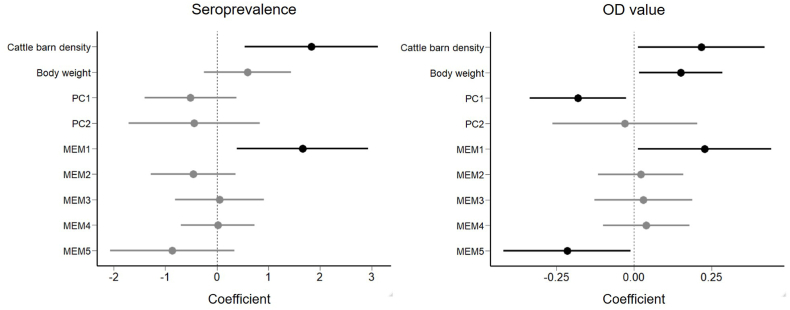
Model averaged coefficients of variables (GLM) explaining seroprevalence and OD values of cats. Bars indicate 95% confidence intervals.

**Fig. 3:**
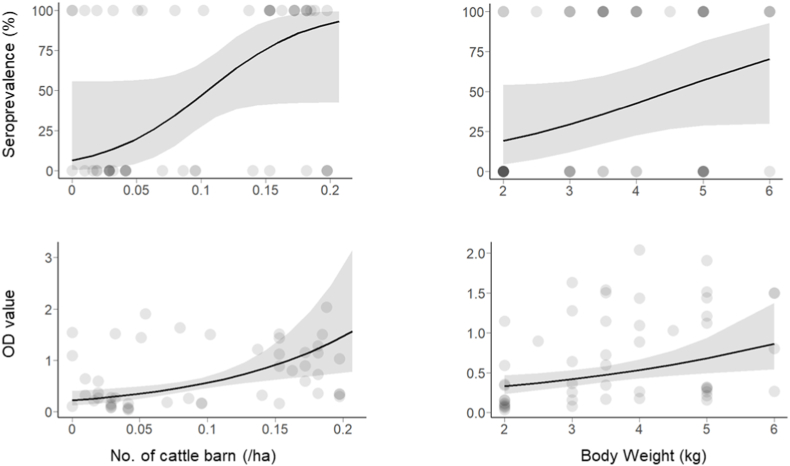
Anti-*Toxoplasma gondii* seroprevalence and OD values of cats as a function of the number of cattle barns within 1 ha or individual body weight. Gray areas indicate 95% confidence intervals. Dots represent individuals. The color becomes darker with increasing sample size.

In cats, we obtained 19 competing models (ΔAIC <2) for seroprevalence and 11 for OD values (Supplementary S1). Model averaging revealed that barn density and MEM1 had a clear influence on seroprevalence, and body weight had a weak influence (Fig. 2); positive coefficients were obtained for barn density and body weight (Fig. 3). The seroprevalence became more than 70% in landscapes with high cattle barn densities. For OD values, model averaging showed that barn density, body weight, PC, and two MEM axes were influential (Fig. 2), with positive coefficients for barn density and body weight (Fig. 3) and negative coefficients for PC1 (i.e., OD values were higher in landscapes with less forests and more residential areas).

**Fig. 4:**
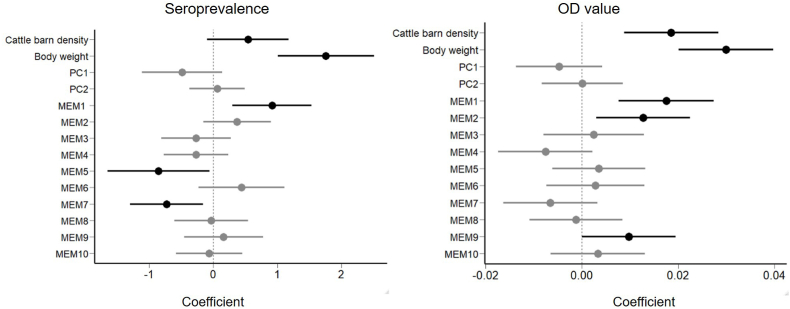
Model averaged coefficients of variables (GLM) explaining seroprevalence and OD values of black rats. Bars indicate 95% confidence intervals.

**Fig. 5:**
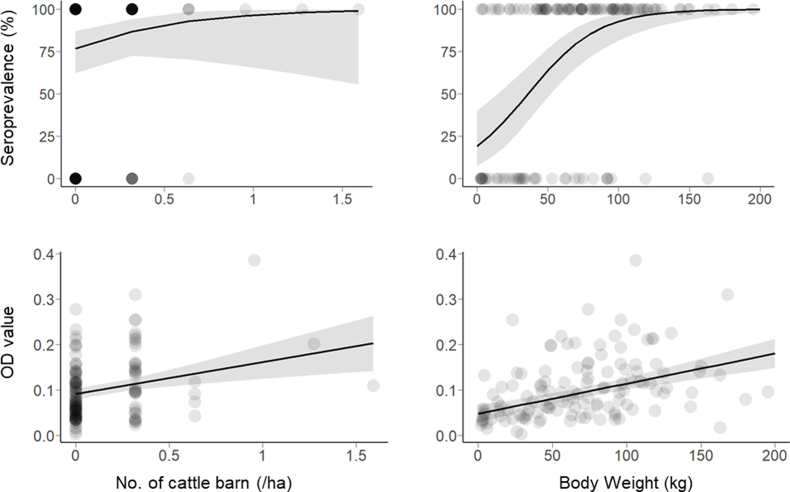
Anti-*Toxoplasma gondii* seroprevalence and OD values of black rats as a function of the number of cattle barns within 1 ha or individual body weight. Gray areas indicate 95% confidence intervals. Dots represent individuals. The color becomes darker with increasing sample size.

In black rats, there were 30 competing models for seroprevalence and 21 for OD values (Supplementary S1). For the seroprevalence model, body weight and the three MEM axes were influential (Fig. 4), with positive coefficients for body weight (Fig. 5). The effect of barn density was weakly positive (Fig. 5), with more than 80% seroprevalence of rats when surrounding cattle barn density was high. In terms of OD values, positive coefficients were obtained for body weight and barn density (Fig. 4, Fig. 5), and three MEMs were also influential (Fig. 4).

### 3.4 Relative rat abundance

**Fig. 6:**
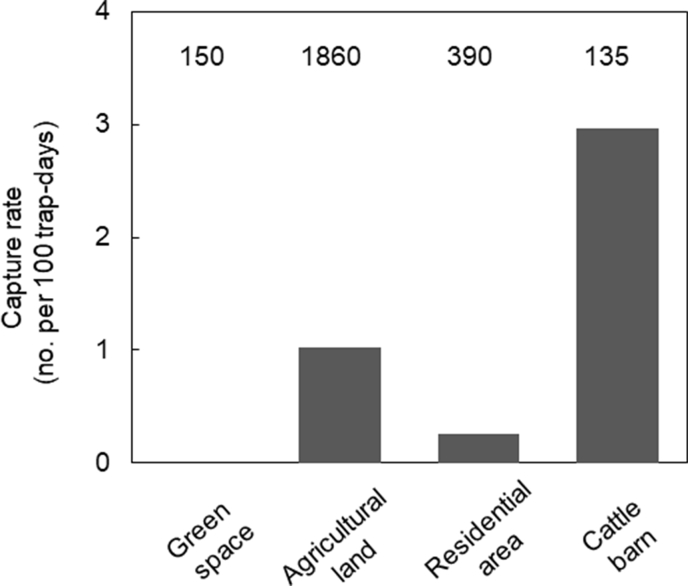
Relative density of black rats in different land-use types, as expressed by the capture rate by traps, i.e., number of individuals captured per 100 trap-days. Numerals above the bars indicate total trap-days.

A total of 155 black rats were captured. Fig. 6 shows capture rate of black rats for different land uses. The capture rate of black rats was significantly higher around barns than in residential areas (Fisher's exact test, P < 0.02) and tended to be higher around barns than in green spaces (P < 0.06).

## 4 Discussion

We investigated antibody levels against *T. gondii* in free-ranging cats and black rats (definitive and representative intermediate hosts) by ELISA and determined the relationships between these levels and landscape environmental factors on Tokunoshima Island, where endangered species are threatened by free-ranging cats. We obtained the following main results. (1) The antibody prevalence and antibody level were high in both cats and black rats in landscapes where the cattle barn density was high. (2) The spatial scale of environmental factors affecting infection was broader in cats than in black rats. (3) Antibody levels in cats tended to be lower in forests and higher around residential areas. This study is one of the few studies clarifying the potential for *T. gondii* transmission from cats to intermediate hosts at the landscape level. Although our study could not link directly the infection status of cats and rats at the same site, we could successfully identify the similarity and difference of the key environmental factors influencing infection risks of *T. gondii* for definitive and intermediate hosts.

The high antibody levels in both cats and black rats in areas with many cattle barns suggest that cattle barns are a hotspot for the maintenance of a high frequency of *T. gondii* infections on Tokunoshima Island. This is consistent with an earlier study showing that the cat density is high in areas with a high barn density on this island (Kazato et al., 2020), which probably caused substantial oocyst shedding by cats in these areas. The high antibody levels and high prevalence of antibodies to *T. gondii* in black rats strongly suggest that *T. gondii* spills over from cats to intermediate hosts via environmental sources, such as soil and water. In addition, our trapping survey indicated that the black rat density is high near cattle barns, likely reflecting the ample food availability derived from compound feed provided to cattle. Thus, the life cycle and sexual reproduction of *T. gondii* may be accelerated by predator–prey interactions between cats and black rats, resulting in high local infection levels (Lélu et al., 2010; Jiang et al., 2012). It is likely that livestock and farmers who frequently come into contact with soil and water in these environments have a greater risk of infection with *T. gondii*. In the future, the possibility of environmental transmission should be tested by detecting *T. gondii* oocyst DNA from soil and water in the field.

Notably, the spatial scale of environmental factors affecting *T. gondii* infection differed between cats and black rats; the best predictive scale was a radius of 1 km for cats and a radius of 100 m for black rats. This difference is reasonable given the difference in home range size between the two species; feral cats have a range of 1–855 ha (e.g., Konecny, 1987; Biró et al., 2004; McGregor et al., 2015; López-Jara et al., 2021), while black rats have a range of less than 1 ha (Byers et al., 2019). Therefore, the effect of cattle barns on *T. gondii* infection was supported by the behavioral characteristics of animals and it is therefore unlikely that this effect was detected simply by chance.

With respect to land use variables, we expected higher infection levels in cats in areas where green space is abundant, where they show a higher density on Tokunoshima Island (Kazato et al., 2020). However, antibody levels were actually lower in areas with more forests and higher around residential areas, as indicated by the negative effect of PC1. This unexpected result might be explained by the lower abundance of black rats in green spaces, as revealed by our capture survey, decreasing the *T. gondii* infection rate in cats. This inference is supported by an extended version of the SIR model showing that cat infection becomes lower with decreasing predation on intermediate hosts (Lélu et al., 2010). On the other hand, the presence of cats with relatively high antibody levels around residential land may reflect the locally high density of domestic cats. However, the effects of forests and residential land were not clearly detected when seroprevalence was used as the objective variable, suggesting that the effects are weak.

We found an overall positive correlation between host body weight and seroprevalence in cats and black rats. This observation has two possible interpretations. First, due to the positive association between age and body weight, heavier individuals inevitably have a greater chance of exposure to *T. gondii* (Reperant et al., 2009; Opsteegh et al., 2012; Gotteland et al., 2014). Second, better nutritional conditions may be related to a higher frequency of contact with *T. gondii*, especially for human-dependent individuals. Since the body weight of cats is nearly saturated in about one year and increases slightly thereafter (Campigotto et al., 2019), body weight may have reflected individual differences in nutritional conditions. Since nutritional conditions may be higher in human-dependent individuals, it is possible that local cat densities increased in these conditions, thereby increasing infection rates.

The positive correlation between OD values and cattle barn density or body weight, especially above the cutoff value, could provide additional information on infection dynamics of *T. gondii* and host animals. Although OD values may be variable with individual physiological conditions after infection, the statistical association of OD values with environmental conditions or body size could be arisen from external factors, such as repeated infections or a progressive decrease in OD values with time since infection. Both processes could produce a temporary transition state in antibody levels, creating a positive association between OD values and exposure to *T. gondii*. In fact, infected wild boars are suspected to regain susceptibility and become re-infected (Opsteegh et al., 2011), although definitive evidence is lacking. It is also speculated that antibody titers in cats may increase as exposure opportunities increase (Afonso et al., 2006) and that the decreased antibody titers in several years after infection is a possible mechanism for the stability of seroprevalence with age in domestic cats (Opsteegh et al., 2012). In the future, it is necessary to experimentally demonstrate whether repeated infections increase antibody levels and whether reversion from infection to susceptibility occurs. If definitive evidence for either of these processes is obtained, the OD value should be more widely used in risk assessments for *T. gondii*.

It is noteworthy that the seroprevalence in cats was around 50%, consistent with previous estimates (e.g., Ballash et al., 2015; Simon et al., 2018), while that in black rats was 70%, which is fairly high for rodents (e.g., Reperant et al., 2009; Gotteland et al., 2014). In theory, seroprevalence in cats should be higher than that in intermediate rodent hosts, as cats are assumed to have antibody to *T. gondii* for a long period (Dubey, 1995), whereas rats are assumed to be infected mainly through contaminated environments (Gotteland et al., 2014). There are three possible explanations for the higher seroprevalence in black rats than in cats, which are not mutually exclusive. First, cats around cattle barns may be well-fed by farmers (Kazato et al., 2020; Qi et al., 2022) and therefore rarely consume black rats, implying that the infection route through intermediate hosts is weakened and the cat seroprevalence may remain low (Afonso et al., 2006). Second, the difference in seroprevalence may be due to the difference in home range sizes between the two animal species. Black rats are likely to have a highly aggregated distribution around cattle barns and a relatively low density across the landscape, as demonstrated by our trapping survey. Moreover, our statistical analysis showed that the spatial scale of infection was an order of magnitude smaller in black rats. Therefore, the contribution of black rats to seroprevalence in cats may be limited to a small spatial scale around cattle barns and is unlikely to be observed at broader spatial scales across landscapes. This means, conversely, that environmental contamination with *T. gondii* is high around cattle barns, conferring a high risk in these areas. Third, a high vertical transmission of black rats in our study area might have caused a higher seroprevalence in this study. High vertical transmissions are reported in natural urban populations of *Mus domesticus* (Marshall et al., 2004), or experimentally infected *Mus musculus* and *Apodemus sylvaticus*, even in chronic infection (Owen and Trees, 1998). Therefore vertical transmission in wild populations of black rats needs further investigation.

Our results revealed that the transmission cycle of *T. gondii* in both the definitive and intermediate hosts is well-established on Tokunoshima Island, although antibody levels were highly heterogeneous across landscapes. More specifically, cattle barns were found to be a hot spot for *T. gondii* infection in both definitive and intermediate hosts. The high density of cats benefiting from feeding cats by farmers can explain this high infection rate (Kazato et al., 2020). Efforts to stop feeding outdoor cats may thus reduce the risk of infection by reducing the cat abundance. Cats are believed to control rats and are widely kept around the world (Coleman and Templ, 1993; Ferreira et al., 2011). Nevertheless, the use of cats for rat control does not necessarily reduce rat abundance (Jackson, 1951; Parsons et al., 2018). Therefore, the use of cats in areas with a high rat abundance may not provide expected benefits and could instead create a situation where the risk of infectious diseases is high. Reducing rat populations may also reduce the prevalence of *T. gondii* infection in cats and eventually livestock and humans by disrupting the infection cycle, as demonstrated by a simulation analysis (Jiang et al., 2012; Deng et al., 2021). Moreover, given the potential vulnerability of native species to toxoplasmosis, such as Amami rabbit (Kubo et al., 2013) and Tokunoshima spiny rat (Tokiwa et al., 2019), the integrated control of cats and rats, both invasive species in most areas of the world, is the key to ecosystem management for native biodiversity, livestock production and human health.

## Authors' contributions

TM, YW, and CS conceptualized this study and TM and YW managed the projects. SO conducted field work and obtained the data. YS analysing seroprevalence of cats and rats with support of YT and CS. SO conducted spatial analysis. SO, YS, CS, YW, TM interpreted the results. SO wrote the original draft with critical revisions by all co-authors. All authors approved the final version for publication and agreed to be accountable for all aspects of this study.

## Data accessibility

The datasets supporting this article have been uploaded as part of the Supplementary S1–S4.

## Ethical statement

This study was approved by the committee for Life Science Research Ethics and Safety in the University of Tokyo (permission number: P21-105) and carried out according to the guidelines of the committee. Also, YW completed the ethical course of Laboratory Animals Module authorized by Association for the Promotion of Research Integrity (course completion report no. AP0000017354).

## Funding statement

YW was funded by the Environment Research and Technology Development Fund of the Environmental Restoration and Conservation Agency of Japan (JPMEERF20184004), and TM was funded by 10.13039/501100001691JSPS KAKENHI Grant Number JP 1K19868.

## Declaration of competing interest

We have no competing interests.
